# Single-cell technologies for multimodal omics measurements

**DOI:** 10.3389/fsysb.2023.1155990

**Published:** 2023-04-21

**Authors:** Dongsheng Bai, Chenxu Zhu

**Affiliations:** ^1^ New York Genome Center, New York, NY, United States; ^2^ Deparatment of Physiology and Biophysics, Institute for Computational Biomedicine, Weill Cornell Medicine, New York, NY, United States

**Keywords:** single-cell, multi-omics, genomics, chromatin states, transcriptome

## Abstract

The recent surge in single-cell genomics, including the development of a wide range of experimental and computational approaches, has provided insights into the complex molecular networks of cells during development and in human diseases at unprecedented resolution. Single-cell transcriptome analysis has enabled high-resolution investigation of cellular heterogeneity in a wide range of cell populations ranging from early embryos to complex tissues—while posing the risk of only capturing a partial picture of the cells’ complex molecular networks. Single-cell multiomics technologies aim to bridge this gap by providing a more holistic view of the cell by simultaneously measuring multiple molecular types from the same cell and providing a more complete view of the interactions and combined functions of multiple regulatory layers at cell-type resolution. In this review, we briefly summarized the recent advances in multimodal single-cell technologies and discussed the challenges and opportunities of the field.

## Introduction

The genome is the blueprint for all living cells and contains the necessary information for the production of RNAs and proteins—the functional molecules responsible for cells’ various biological processes. Genomic analyses, including profiling the variations in genome sequences, the epigenetic states of regulatory elements, and the abundance of RNAs, have provided insights into the fundamental principles that govern the molecular processes of the cell. However, analyzing a mixture of cell populations results in averaged signals of various cell types, obscuring the cell-to-cell variations in molecular states—which are crucial for the proper biological functions in multicellular organisms. Recent advances in single-cell genomics enabled the dissection of cell-type-specific molecular programs from complex cellular environments by isolation and analysis of individual cells (Papalexi and Satija, 2018). For example, the dynamic transcriptional programs are tightly linked to cells’ identity and functional states; analyzing transcriptome at the single-cell level allows the interrogation of shared and distinct expression programs of cells (Tang et al., 2009). DNA methylation is an epigenetic mark that regulates and maintains cell-type-specific transcriptional programs. The analysis of DNA methylome from single cells enabled the assessment of the epigenetic heterogeneity (Smallwood et al., 2014). Cell-type-specific regulatory elements are associated with chromatin accessibility, and different combinations of histone modifications lead to activation or repression of genes; modules of coordinated regulatory elements could be identified by single-cell measurement of chromatin states (Buenrostro et al., 2015; Cusanovich et al., 2015; Rotem et al., 2015). High-order chromosome structures have been linked to gene regulation and DNA replication and repair. The association of chromosome territory structure variabilities with genome activation patterns was revealed by capturing of chromosome structure in single cells (Nagano et al., 2013).

**FIGURE 1:**
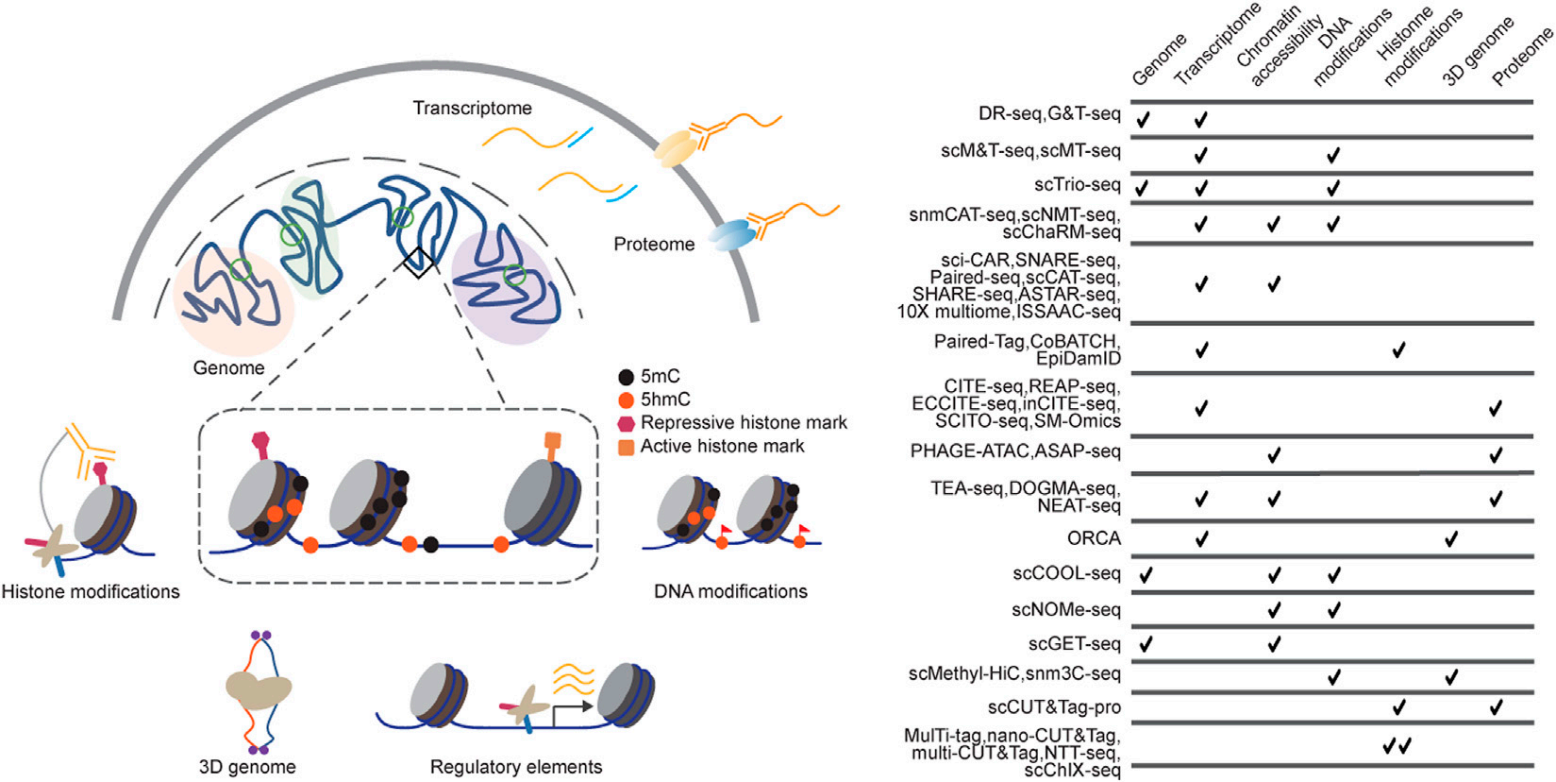
Technologies for single-cell multimodal measurements. **(A)** Single-cell multi-omics methods have been developed to profile DNA sequences, chromatin states, and gene expression from the same cells. Single-cell multimodal omics methods are designed on various strategies, including Tn5 tagmentation, proximity ligation, immunoprecipitation, and chemical labelling, among which they perform high biocompatibility and orthogonality; **(B)** the table summarizes some of the current single-cell multi-omics sequencing methods.

However, measuring one modality at a time only captures the partial picture of the complex molecular network, while the intricate interactions and combined effects from multiple molecular layers could be lost. Therefore, measuring multiple molecular layers jointly from the same cells is crucial for gaining a more complete understanding of these complex molecular programs of the cells. Building on stand-alone single-cell genomics technologies, single-cell multimodal omics approaches have recently been developed to perform multiple measurements from the same cells (Figure 1). Computational approaches were also developed to perform multimodal integration of associated measurements to identify the relationships and crosstalk between multiple variables, enhancing our understanding of the determination and maintenance of cellular states (Chappell et al., 2018; Efremova and Teichmann, 2020; Zhu et al., 2020).

### Linking genome with transcriptome

Genomic variation is the differences in DNA sequence among individuals or populations and could contribute to abnormities, such as anosmia and complex neurological diseases (Hasin-Brumshtein et al., 2009; Zhang et al., 2009). Joint analysis of genome and transcriptome enables a better understanding of how genetic variations affect the behaviors of individual cells and therefore holds great promise for understanding the biology of cancers (Group et al., 2020). Build on single-cell WGA (whole genome amplification) (Zong et al., 2012) and RNA sequencing methods (Hashimshony et al., 2012), DR-seq (gDNA-mRNA sequencing) (Dey et al., 2015) and G&T-seq (genome and transcriptome sequencing) (Macaulay et al., 2015) have been developed to profile the genome variation and transcriptome simultaneously. The relationships of chromosomal aneuploidies and interchromosomal fusions with the variabilities of gene expression among individual cells were revealed by these analyses at single-cell resolution. Additionally, these methods could be used for the assessment of the origin and evolution of tumors by lineage tracing with enhanced sensitivity via tracking both genomic DNA and massager RNA at the single-cell level (Wagner and Klein, 2020).

### Chromatin states and gene expression

Chemical modifications on DNA bases, such as cytosine methylation (5 mC), can alter the activity states of cis-regulatory elements (CREs) by facilitating or inhibiting the binding of transcription factors to CREs in a cell type-specific manner (Smith and Meissner, 2013). DNA modifications are associated with various biological processes, including development, disease, and aging (Greenberg and Bourc’his, 2019). Based on the optimized bisulfite treatment to achieve base-resolution detection of 5 mC from a limited amount of input materials, methods for single-cell parallel profiling of the methylome and transcriptome have been developed to dissect the relationships between gene expression and 5 mC modification levels in functional genomic regions (Angermueller et al., 2016; Hou et al., 2016; Hu et al., 2016; Luo et al., 2022). ScTrio-seq further links genomic copy-number variations with DNA methylome and transcriptome in individual cells and dissects the regulatory heterogeneity in hepatocellular carcinomas (Hou et al., 2016). However, these methods depend on the physical separation of cytoplasm RNA and nucleic genome DNA (gDNA) and run into the risk of material loss. The recently developed snmCAT-seq incorporates 5-methyl-dCTP during reverse transcription of RNA—the resulting cDNA are resistant to bisulfite treatment, and thus, the “C-to-T” signal could be used to identify genome DNA fragments from cDNA derived from mRNA without the need of separating mRNA and gDNA prior to amplification (Luo et al., 2022). Their integrative approach enabled the reconstruction of the regulatory hierarchy for 63 human cortical cell types and the prediction of cell types that are associated with diseases.

To identify the location of CREs, assays including DNase-seq (Boyle et al., 2008), MNase-seq (Schlesinger et al., 2013), and ATAC-seq (Buenrostro et al., 2013) were developed to profile chromatin accessibilities. ATAC-seq uses the hyperactive Tn5 transposase to perform simultaneous fragmentation and tagging of open chromatin regions for subsequent library amplification and sequencing. Based on this streamlined sample-to-sequencing library workflow, single-cell ATAC-seq has been developed and widely used to map candidate cis-regulatory elements to resolve cell type-specific usage of “regulatome” (Cusanovich et al., 2015; Mezger et al., 2018; Lareau et al., 2019). Combining scATAC-seq with various single-cell indexing platforms, including combinatorial barcoding and microfluidics systems, high-throughput methods for joint analysis of chromosome accessibility and transcription were developed to reveal the associations between regulatory elements and putative targeted genes. These methods demonstrated their utilities in gaining a global view of the cellular composition and cell type-specific molecular programs in healthy tissues and diseases by the parallel generation of epigenome and transcriptome maps from a large number of single cells (Cao et al., 2018; Lake et al., 2018; Chen et al., 2019; Liu et al., 2019; Zhu et al., 2019; Ma et al., 2020; Xu et al., 2022). For example, sci-CAR and Paired-seq linked distal CREs to potential target genes with the covariance of chromatin accessibility and transcription across single cells (Cao et al., 2018; Zhu et al., 2019); SHARE-seq measures the chromatin potential of single cells and enables the finer dissection of closely related cellular states compared to scRNA-seq (Ma et al., 2020).

The active or repressive roles of regulatory elements are defined by their epigenetic states, such as different combinations of histone modifications and binding of transcriptional factors. Chromatin immunoprecipitation followed by sequencing (ChIP-seq) is the gold standard for analyzing chromatin-protein interactions, while the requirement for a large amount of input materials makes single-cell ChIP-seq analysis challenging (Rotem et al., 2015). CUT&Tag based on *in situ* tagmentation guided by antibodies targeting histone modifications or transcription factors of interest significantly reduced the input requirements and enabled the investigation of histone modifications at the single-cell level (Kaya-Okur et al., 2019; Bartosovic et al., 2021; Wu et al., 2021). However, different histone mark datasets may have varying resolutions in separating cell groups - due to both technical factors (such as antibody efficiencies and specificities) and their distinct biological functions (such as their genome distribution patterns and relationships with gene expressions), and thus computational integration of multiple different histone modification datasets is challenging. Jointly profiling of histone modifications with gene expression could allow unbiased integration of different chromatin states datasets using transcriptome as the central modality (Xiong et al., 2021; Zhu et al., 2021; Rang et al., 2022). Such strategies could also be applied to predict the putative target genes for both the active and repressive regulatory elements at cell-type resolution in complex tissues (Zhu et al., 2021).

### Towards higher-order chromosome organization

In addition to the epigenetics states of regulatory elements, the three-dimensional organization of the genome also makes key contributions to the regulation of gene expressions. Chromosome conformation capture sequencing methods were developed to map the interactions between distal chromatin regions and provided insights into how the genome is organized and how they regulate gene expression programs (Lieberman-Aiden et al., 2009). Single-cell Hi-C techniques capture the snapshots of 3D genome organization 1 cell at a time and reveal the dynamic and distinct chromosome conformations during different cell cycle phases and cell types (Nagano et al., 2013; Ramani et al., 2017; Stevens et al., 2017). However, the chromosome conformation profiles do not directly reflect the transcriptional or translational states of cells, and thus identifying and annotating the cell identities from the single-cell Hi-C datasets alone is challenging. To overcome this barrier, methods for co-capturing chromosome conformations with other modalities, such as transcription and DNA methylation in single cells, were developed (Lee et al., 2019; Li et al., 2019; Mateo et al., 2019). Combining RNA-fluorescence *in situ* hybridization (RNA-FISH) with parallel visualizing of DNA folding in single-cells, ORCA (optical reconstruction of chromatin architecture) revealed physical borders between active and repressed DNA exist in a cell type-specific manner in *Drosophila* cells (Mateo et al., 2019). Joint analysis of DNA methylation and chromosome conformation revealed coordinated DNA methylation states between distal genomic regions that are in spatial proximity and allowed reconstruction of cell type-specific chromatin organization of complex tissues (Lee et al., 2019; Li et al., 2019).

### From RNA to proteins

The transcriptome of cells could function as a central modality for integrating multiple different molecular layers in single-cell multiomics analyses. However, transcriptome analysis only provides information on genes that are being transcribed, but not the abundance of the cell’s functional molecule proteins, and analyzing the functional states of individual cells requires measuring the protein abundances directly. Recently, cellular barcoding of epitopes and transcriptomes emerged as a promising technique for analyzing cell surface proteins of single cells together with transcriptomes (Peterson et al., 2017; Stoeckius et al., 2017). In these methods, cells were incubated with the barcode oligo-conjugated antibodies targeting a panel of cell surface proteins, followed by micro-fluidics capturing both the antibody barcode oligos and mRNA from thousands of single cells in parallel. The cell surface protein abundances can also be jointly measured with histone modifications: by combining CUT&Tag with CITE-seq, scCUT&Tag-pro measures cell surface protein abundances together with histone modifications from the same cell at a time, and scChromHMM integrates different histone modifications use protein abundances as the central modality to generate single-cell “megaomic” profiles, enabling the exploration of heterogeneity in chromatin state across discrete cell types and continuous trajectories (Zhang et al., 2022). These approaches could be further combined with capturing chromatin accessibilities for integrating the epigenome, transcriptome, and protein, providing more complete views on gene regulation (Mimitou et al., 2021; Swanson et al., 2021; Chen A. F. et al., 2022).

### Measuring multiple epigenome layers

Another frontier in single-cell multiomics is to dissect the relationships between different regulatory layers and their combined effects on cell function. The combinations of transcription factors and CREs define cell types and developmental trajectories of multicellular organisms. To map multiple chromatin features from the same cells at a time, MulTI-Tag and multi-CUT&Tag pre-barcoded the antibody-tethered transposases targeting multiple chromatin-associated proteins and multiplex them for immunostaining of the cells (Gopalan et al., 2021; Meers et al., 2022). Such analyses resolved unique and coordinated patterns of active and repressive regulatory element usages in distinct cell types and states and allowed analysis of the direct interaction between different chromatin-associated proteins. NTT-seq and nano-CUT&Tag used an alternative strategy of harnessing nanobody-tethered transposases specifically targeting different immunoglobulin-G contain antibodies to pre-barcode antibody-transposase complexes, for the multiplexed chromatin-associated proteins detection (Bartosovic and Castelo-Branco, 2022; Stuart et al., 2022). NTT-seq was also extended for joint profiling of cell surface protein abundances with multifactorial chromatin states, which is particularly useful for understanding the dynamic gene regulation programs in the immune systems (Stuart et al., 2022). By engineering transposase to include the chromodomain of heterochromatin protein-1α, scGET-seq comprehensively assays open and closed chromatin regions and deduced Chromatin Velocity to uncover epigenetic reorganization paths during stem cell reprogramming (Tedesco et al., 2022). The distinct distribution patterns of certain combinations of chromatin-associated proteins could also be utilized to deconvolute their relationships in the same cells. CUT&Tag2for1 obtained the combined signal from H3K27me3 and Pol2S5p and computationally deconvoluted the signals to give high-resolution maps of both the active and repressive regulomes in single cells (Janssens et al., 2022). Benefited from the higher-resolution footprints generated by antibody-tethered MNase, scChIX-seq was able to multiplex and deconvolute additional combinations of chromatin-associated proteins, including H3K27me3/H3K9me3, H3K4me1/H3K27me3, and H3K36me3/H3K9me3 (Yeung et al., 2023).

### Multimodal genomics analysis with spatial information

Single-cell multiomics sequencing tools have increased our ability to study the gene regulation mechanisms in tissues; however, the spatial context is lost during tissue dissociation in such analyses. The crosstalk between cells in their native microenvironment is critical for the understanding of the complex cellular networks in health and diseases. To overcome this barrier, a suite of spatial technologies was recently developed for joint analysis of genome, transcriptome, epigenome, and protein abundances. Built on microscopy approaches, optical reconstruction of chromatin architecture (ORCA) linked visualization of DNA folding with RNA in single cells (Mateo et al., 2019) and revealed cell differentiation is associated with extensive 3D remodeling of chromatin structure in developmental control loci. Based on *in situ* capture of cDNA and antibody tags, SPOTS (Ben-Chetrit et al., 2023) and SM-Omics (Vickovic et al., 2022) performed simultaneous epitope and transcriptome profiling in single cells and enhanced the analysis of differential gene expression programs across tissue regions. By introducing spatial information to single cells with a deterministic barcoding approach, spatial-ATAC-RNA-seq, and spatial-CUT&Tag-RNA-seq enabled co-profiling of accessible chromatin or histone modifications with transcriptome on the same tissue section and uncovered new biological insights in epigenetic priming and gene regulation at different regions of the tissues (Zhang et al., 2023).

## Discussions

Single-cell multiomics technologies simultaneously measure multiple types of molecular layers from the same cells, including genome, epigenome, transcriptome, and proteome. These approaches can provide a more comprehensive understanding of the underlying molecular mechanisms governing cellular diversity and function. However, single-cell multiomics is still in its infancy, and several challenges need to be addressed to fully unleash the potential in providing systems understanding of cell’s molecular networks and become more widely adopted by the research community. One key challenge for single-cell multiomics is the balance between data sparsity and throughput. Many current single-cell multiomics approaches are still in the early stages of development and may not be able to capture all the desired molecular layers with high selectivity and sensitivity. The coverages of epigenome and transcriptome for individual cells provided by current high-throughput methods are still low, rendering it difficult to identify cell-to-cell variability from technical noise. Methods for analyzing individual cells could provide much higher sensitivity by saturated measurement, while profiling limited numbers of cells could result in biased representations of the global cellular population. While optimizing existing experimental procedures may help to minimize the gaps, new biochemical methods and approaches may be needed to completely overcome this limitation. The recent drop in sequencing costs has greatly facilitated the generation of single-cell multiomics datasets, and the large-scale analysis and integration of datasets generated by single-cell multiomics technologies have become another major challenge, and future development of computational and bioinformatic methods and tools are also desired (Efremova and Teichmann, 2020). Another area of single-cell multiomics that is less developed is the examination of cellular program changes over time. The use of metabolic labeling to measure newly synthesized mRNA in individual cells was employed to quantitatively analyze the changes in transcription within complex systems (Cao et al., 2020; Qiu et al., 2020). It is likely that this approach could also be adapted to investigate the dynamics of molecular networks during the determination and transition of cell states. An alternative strategy is to simultaneously measure related molecular events, such as the “writing” and “erasing” of epigenetic modifications, to model the forward and reverse rates of cellular reprogramming and to reveal the underlying principles guiding cell state transition, especially during the initiation and progression of human diseases. Finally, future developments for the measurement of multiple molecular layers of single cells without compromising the cell’s viability (Chen W. et al., 2022) could uncover the underlying molecular mechanisms of various cellular processes by linking the initial state of molecular networks with downstream responses.

The field of single-cell multiomics holds great potential and is continually advancing with new experimental and computational approaches to overcome its current limitations. Single-cell multiomics could provide opportunities to identify new therapeutic targets and biomarkers for precision medicine by obtaining comprehensive information on multiple modalities simultaneously. By dissecting the interactions and crosstalk between multiple components within the same cells, single-cell multiomics could also provide insights into the molecular principles for disease establishment and progression and shed light on the future development of treatments.
